# LepVax, a defined subunit vaccine that provides effective pre-exposure and post-exposure prophylaxis of *M. leprae* infection

**DOI:** 10.1038/s41541-018-0050-z

**Published:** 2018-03-28

**Authors:** Malcolm S. Duthie, Maria T. Pena, Gigi J. Ebenezer, Thomas P. Gillis, Rahul Sharma, Kelly Cunningham, Michael Polydefkis, Yumi Maeda, Masahiko Makino, Richard W. Truman, Steven G. Reed

**Affiliations:** 1 Infectious Disease Research Institute, 1616 Eastlake Ave E, Suite 400, Seattle, WA 98102 USA; 2 National Hansens Disease Programs, Baton Rouge, LA USA; 3 Department of Neurology, Johns Hopkins University, Baltimore, MD 21209 USA; 4 Department of Microbiology, Immunology and Parasitology, LSU School of Medicine, New Orleans, LA USA; 5 Department of Mycobacteriology, Leprosy Research Center, National Institute of Infectious Diseases, Tokyo, Japan; 6 Department of Pathobiological Sciences, School of Veterinary Medicine, Louisiana State University, Baton Rouge, LA USA

## Abstract

Sustained elimination of leprosy as a global health concern likely requires a vaccine. The current standard, BCG, confers only partial protection and precipitates paucibacillary (PB) disease in some instances. When injected into mice with the T helper 1 (Th1)-biasing adjuvant formulation Glucopyranosyl Lipid Adjuvant in stable emulsion (GLA-SE), a cocktail of three prioritized antigens (ML2055, ML2380 and ML2028) reduced *M. leprae* infection levels. Recognition and protective efficacy of a single chimeric fusion protein incorporating these antigens, LEP-F1, was confirmed in similar experiments. The impact of post-exposure immunization was then assessed in nine-banded armadillos that demonstrate a functional recapitulation of leprosy. Armadillos were infected with *M. leprae* 1 month before the initiation of post-exposure prophylaxis. While BCG precipitated motor nerve conduction abnormalities more rapidly and severely than observed for control infected armadillos, motor nerve injury in armadillos treated three times, at monthly intervals with LepVax was appreciably delayed. Biopsy of cutaneous nerves indicated that epidermal nerve fiber density was not significantly altered in *M. leprae*-infected animals although Remak Schwann cells of the cutaneous nerves in the distal leg were denser in the infected armadillos. Importantly, LepVax immunization did not exacerbate cutaneous nerve involvement due to *M. leprae* infection, indicating its safe use. There was no intraneural inflammation but a reduction of intra axonal edema suggested that LepVax treatment might restore some early sensory axonal function. These data indicate that post-exposure prophylaxis with LepVax not only appears safe but, unlike BCG, alleviates and delays the neurologic disruptions caused by *M. leprae* infection.

## Leprosy: Working towards a safe and effective vaccine

A leprosy vaccine candidate has been developed that raises immune responses against targets gleaned from naturally resistant individuals. Researchers from the United States and Japan, led by Malcolm Duthie, of Seattle’s Infectious Disease Research Institute, tested a *Mycobacterium leprae* vaccine candidate that generated immune responses mimicking those found in partially-resistant patients, and immune co-inhabitants of the severely infected. The candidate, dubbed LepVax, inhibited infection in mice and, when administered post-infection, delayed and mitigated nerve damage in armadillos. This contrasts with the current vaccine, BCG, which can precipitate leprosy symptoms when given after infection. This study also revealed that *M. leprae* infection can induce ‘silent’ pre-clinical nerve aberations. High-risk populations may already be infected with *M. leprae*, making safe and effective post-exposure prophylaxis a landmark step in combating both the individual and global burden of leprosy.

## Introduction

Leprosy (Hansen’s disease) is a dermatological and peripheral neurological disorder caused by *Mycobacterium leprae* infection that presents as skin lesions and sensory and/or motor neuron damage. It is among the leading causes of non-traumatic peripheral neuropathies worldwide.^1,2^ Multidrug therapy (MDT) has been distributed free of charge for registered leprosy patients on a world wide scale in the drive toward “elimination” of leprosy as a global health problem. Declines in global incidence prompted by the MDT program have now levelled off and recent new case incidence rates indicate re-emergence in many regions that previously reported elimination. Transmission of *M. leprae* therefore continues and, worryingly, it is widely believed that a large number of cases go unreported.^3^

In addition to provision of MDT for patients, the current pursuit of preventative measures against leprosy involves chemoprophylaxis (single-dose rifampicin; SDR) within high-risk populations. This strategy has limitations as SDR has previously provided only a transient (2–3 years) abatement in new case detection rates.^4–6^ Unlike drug treatment vaccines provide active and sustained protection by promoting immune memory responses.^7–9^ While immunization with *M. bovis* Bacillus Calmette–Guérin (BCG) provides some protection, meta-analyses of clinical trials indicating a modest ability to prevent leprosy (26% for observational and 41% for experimental studies, respectively) and the persistence of leprosy in regions with good BCG coverage highlight that additional strategies are required.^7,10^ Multibacillary (MB) leprosy patients present with many disseminated skin lesions and large bacterial burdens, indicating that the strong humoral immune responses that they classically exhibit are not protective. In contrast, replication and dissemination of *M. leprae* is limited in PB leprosy patients, and despite presumed exposure to *M. leprae*, the vast majority of healthy household contacts (HHC) of MB patients appear to develop effective immunity. Thus, antigens that are recognized by PB patients or HHC are believed to be targets of an effective immune response against *M. leprae*.

The consequence of immunization on *M. leprae*-associated neuropathy has not been investigated in a controlled experimental model. Experimental *M. leprae* infection in nine-banded armadillos (*Dasypus novemcinctus*) closely recapitulates many of the structural, physiological, and functional aspects of MB (lepromatous) leprosy.^11–18^ Inflammation and demyelinating neuropathy are observed during histopathological inspection of infected nerves and a nerve function deficit can be detected using electrophysiology.^11,12^ The progression of small sensory neuropathies can be determined by immunohistochemical staining, alongside morphological and quantitative assessment, of small sensory fibers in skin punches.^19–22^

The current investigation used a reverse vaccinology approach to develop a defined subunit vaccine capable of reducing *M. leprae* burden when provided prophylactically to mice. We then used cutting edge neurological assays to assess the impact of post-exposure immunization on peripheral and cutaneous small sensory fibers, with particular regard to immune-mediated nerve damage, during experimental infection in armadillos. Our data indicate the development of a safe vaccine with the potential to limit the progression of *M. leprae* infection to leprosy.

## Results

### Immunization with select antigens reduces M. leprae infection

**Fig. 1:**
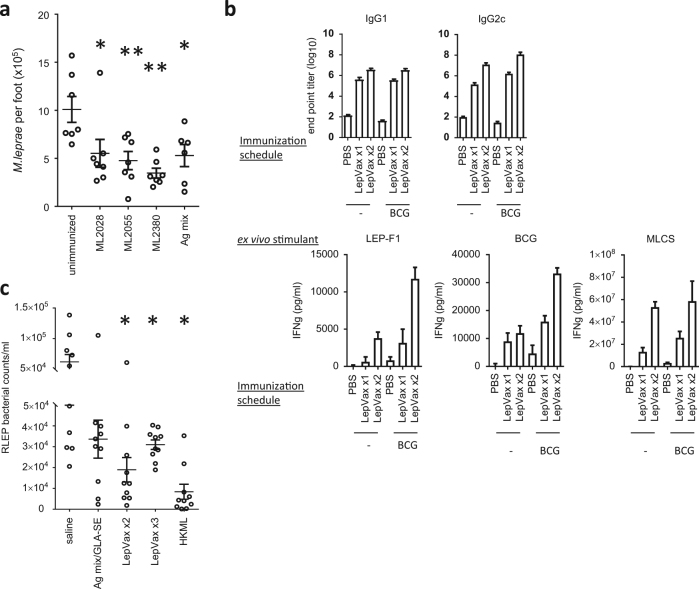
Induction of protective anti-*M. leprae* responses by recombinant antigens formulated in GLA-SE. In **a** mice were injected subcutaneously with antigens/GLA-SE at biweekly intervals, for a total of three immunizations. One month after the last immunization mice were infected with 1 × 10^4^ *M. leprae* in each foot, and bacterial burdens determined 12 months later. Results are shown as mean and s.e.m. Mann–Whitney test was used to calculate *p*-values between each group; *n* = 7 per group. In **b**, mice were injected, or not, with BCG vaccine then 1 month later were subsequently injected subcutaneously with LepVax (at biweekly intervals if immunized more than once). Serum and spleens were collected 1 month after the final immunization to determine antigen-specific immune responses Top panel, LEPF1-specific serum IgG1 and IgG2c titers were determined by ELISA. Middle and bottom panels, single-cell suspensions were prepared from each spleen and incubated with 10 μg/ml indicated protein (BCG = BCG lysate; MLCS = *M. leprae* cell sonicate), then culture supernatants collected 72 h later and IFNγ content determined by ELISA. Responses were corrected by the subtraction of the IFNγ concentration observed in the wells of unimmunized mice. Results are shown as mean and s.e.m; *n* = 5 per group. Data are representative of two independent experiments. In **c**, mice were immunized with a mixture of GLA-SE and either recombinant antigens represented in LepVax or with LepVax (either two or three times at biweekly intervals), or with heat-killed *M. leprae* (HKML) alone (no exogenous adjuvant added). One month after the last immunization mice were infected with 1 × 10^4^ *M. leprae* in each foot, and bacterial burdens determined 12 months later. Results are shown as mean and s.e.m. Mann–Whitney test was used to calculate *p*-values between each group; *n* = 10 per group. *p-value < 0.05 and **p-value < 0.01 versus unimmunized control

Replication and dissemination of *M. leprae* is limited in PB leprosy patients and most HHC, suggesting that antigens that they recognize are potentially targets of an effective immune response against *M. leprae*. We previously identified several antigens that fit this criterion.^23,24^ To investigate if immunization with the antigens ML2028, ML2055 and ML2380 could limit *M. leprae* infection, mice were immunized with single antigens, or combinations of antigens, formulated with GLA-SE, a Toll-like receptor (TLR) 4 ligand in stable emulsion (SE). Following infection with *M. leprae* significantly fewer bacteria were recovered from the footpads of immunized mice relative to the numbers recovered from unimmunized control mice (Fig. 1a; *p*-values <0.05). These data indicate that immunization with the selected antigens elicits responses that protect mice against *M. leprae* challenge and supports their inclusion within a defined sub-unit vaccine against leprosy.

While also increasing the proportion of the population that is likely to respond, combining multiple antigens into a single fusion protein is now commonly used to provide a more consistent production process. We therefore created a single tetravalent 89kD fusion protein, designated LEP-F1, consisting of the ML2028, ML2055 and ML2380 antigens, with the addition of ML2531 to stabilize expression. When mice were immunized with LEP-F1 in conjunction with GLA-SE (LepVax) they raised antibodies against each component indicating that the antigenicity of each component was retained in the fusion (data not shown). Given that the BCG vaccine is routinely used in leprosy-affected regions, we also examined if prior BCG immunization led to any interactions upon LepVax immunization. Mice were either primed, or not, with BCG then immunized with LepVax. Subsequent analyses of the IFNγ recall response to LEP-F1 indicated antigen-specific responses following either immunization scheme (Fig. 1b). Furthermore, mice immunized with LepVax also responded to lysate of BCG and, most importantly, to crude *M. leprae* antigens (Fig. 1b and data not shown). These data indicate that not only that immunization with Lep Vax raises responses that recognize *M. leprae*, but that these responses are not adversely affected by prior BCG immunization.

### Immunization with LepVax reduces M. leprae burdens

We hypothesized that immunization with LepVax would limit bacterial growth, and therefore evaluated its ability to protect against experimental *M. leprae* infection. Mice were immunized then infected with *M. leprae* in the footpad. When assessed 12 months later the bacterial burdens of mice immunized with LepVax were approximately 85% lower than those observed in mice that were injected with the GLA-SE adjuvant formulation alone (Supplementary Figure 1; *p*-value < 0.05). Immunization with LepVax elicited protection equivalent to the mixture of its individual components, and provided protection when injected two or three times (Fig. 1c; *p*-values <0.05 versus unimmunized). Statistical analyses between the 2× and 3× LepVax-immunized groups, and the 2 × LepVax and HKML-immunized groups, indicates that they are not significantly different (*p*-values = 0.07 and 0.14, respectively), but the 3 × LepVax and HKML-immunized groups are (*p*-value < 0.01). The reason for this vagrancy is unclear. Irrespective of this, our experimental data indicate that the defined subunit LepVax vaccine induces immune responses that significantly limit *M. leprae* infection.

### Immunization with LepVax delays motor nerve function impairment

**Fig. 2:**
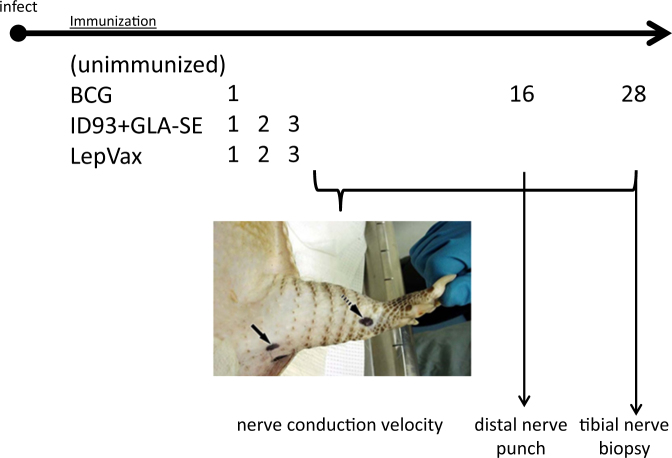
Experimental set-up to examine post-exposure immunoprophylaxis in *M. leprae*-infected armadillos. Armadillos underwent intravenous inoculation with *M. leprae*, then 1 month later were: left untreated (unimmunized); immunized with BCG (BCG) one time; immunized with ID93 + GLA-SE, for a total of three times at monthly intervals or; immunized with LepVax (LEP-F1 + GLA-SE), for a total of three times at monthly intervals. The animals were monitored for compound muscle action potential (CMAP) and motor nerve conduction velocity (NCV) at monthly intervals, underwent three mm skin punches 16 months, and had their posterior tibial nerves (TN) biopsied at 28 months, after *M. leprae* inoculation. The photograph is the authors own

**Fig. 3:**
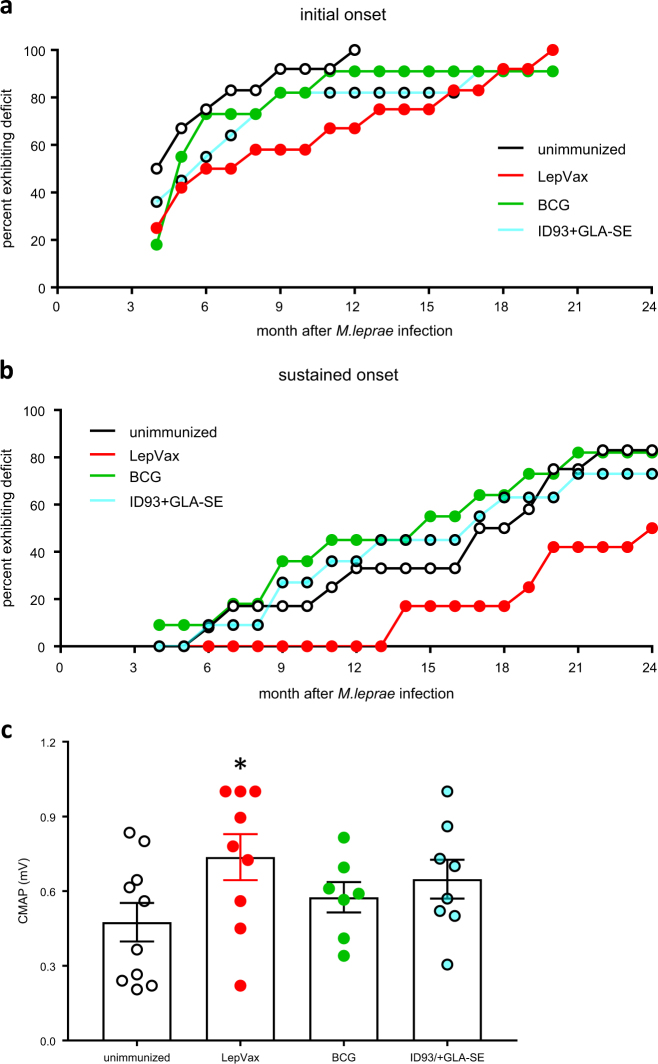
Immunization with LepVax delays *M. leprae*-induced motor nerve damage. Armadillos were monitored for compound muscle action potential (cMAP) and motor nerve conduction velocity (NCV) at monthly intervals following infection and immunization. In **a**, the first month at which each animal first showed an abnormal nerve conduction was recorded and in **b** the time at which three consecutive abnormal readings were obtained for each particular animal is noted. The cumulative percent for each group is shown, and data shown are from one of two similar experiments. In **c**, CMAP measurements from 24 months after infection are shown. Each point depicts the data from a single armadillo, while the box and vertical bars show the mean and s.e.m, respectively. *p-value < 0.05 versus unimmunized, infected control animals

Many clinicians fear that generation of an inflammatory immune response by vaccination will precipitate leprosy, or leprosy reactions, in infected individuals. Given that the hallmark of leprosy is nerve damage, we evaluated the impact of immunization in nine-banded armadillos. To mimic asymptomatic *M. leprae* infection, a situation that is likely common in leprosy hyper-endemic regions, armadillos were first infected, then immunized and monitored (Fig. 2). Infected, but untreated armadillos, began to show nerve conduction deficits as early as 4 months after inoculation, and all of the control armadillos had exhibited at least some measurable deficit by 12 months (Fig. 3a). We noticed that in the early stages of disease development conduction deficits could fluctuate and be transient. To account for these fluctuations, we also assessed onset of sustained conduction deficit as defined by exhibiting abnormal readings for three consecutive months. The variable nature of *M. leprae* infection in these outbred animals became apparent using this parameter, with sustained nerve conduction deficits occurring over a wide range of 6–22 months after infection and 2 of 12 (17%) infected armadillos not actually demonstrating persistent alterations (Fig. 3a). In comparison to previously established compound motor action potential (CMAP) amplitude data in uninfected armadillos (0.9 mV ± 2 SD),^13^ a lower CMAP was observed among *M. leprae*-infected animals and this continued to decline over time. Interestingly, BCG immunization of already infected animals led to precipitation of nerve damage. While onset of conduction deficits in BCG vaccinated armadillos occurred at the same time as control untreated animals (Fig. 3a), sustained conduction deficits were more rapidly observed in BCG-vaccinated armadillos than control untreated animals (Fig. 3b). The extent of the dissemination was significant enough that 27% (3 of 11) of the BCG immunized armadillos had to be removed from the study. In stark contrast, LepVax immunization delayed the onset of motor nerve conduction abnormality and reduced the proportion of animals that developed sustained damage (Fig. 3a, b). The CMAP showed a moderate, but significant, improvement in LepVax immunized animals 24 months after infection (Fig. 3c; *p*-value < 0.05). The improvement generated by LepVax immunization was not be attributable to an adjuvant impact because armadillos treated with the tuberculosis vaccine ID93 + GLA-SE did not exhibit any benefits (Fig. 3a, b). Taken together, these data reveal that LepVax immunization of infected armadillos delayed and alleviated the *M. leprae*-induced motor nerve damage. Thus, when provides in a post-exposure setting, unlike BCG, LepVax delays and alleviates *M. leprae*-induced motor nerve damage.

### Enumeration and physical characterization of tibial nerves

**Fig. 4:**
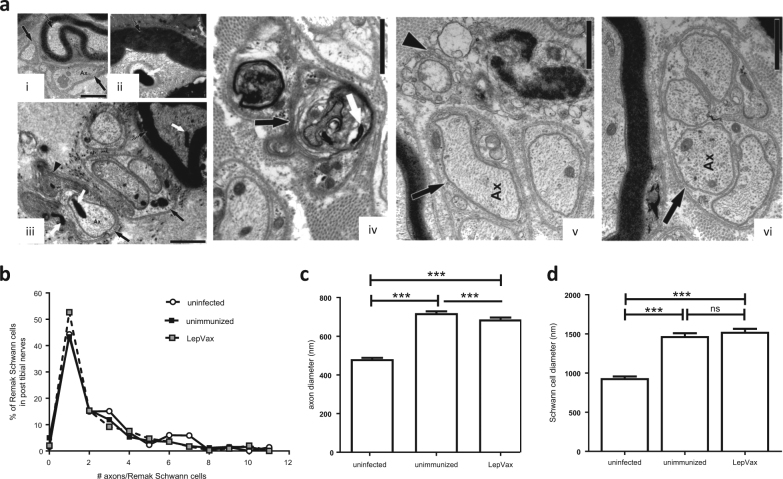
Physical characterization and morphometry of tibial nerves in armadillos. Armadillos were infected with *M. leprae* then had their posterior tibial nerves biopsied 28 months later. In **a** electron micrographs of a tibial nerve were taken. In (i), both Remak bundles (arrows) and myelinated axon are observed in nerves collected from an uninfected animal, with a Remak axon containing a cluster of mitochondria indicated (Ax). In (ii), a myelinated axon from a *M. leprae*-infected armadillo (broken arrow) with a *M. leprae* bacillus (white arrow) bounded by vacuolated axoplasm is shown. In (iii), a microphotograph of a *M. leprae*-infected tibial nerve section containing Remak bundles (solid black arrows) and a myelinated axon (broken black arrow) can be seen, along with *M. leprae* in axoplasm and Schwann cell cytoplasm (white arrows) exhibiting edema and degeneration (arrow head). In (iv) a Remak Schwann cell (black arrow) with *M. leprae* (white arrow) undergoing degeneration is identified. In (v), nerves from a LepVax-immunized armadillo showed an occasional intraneural macrophage (arrow head) adjacent to Remak axons (arrow), and in (vi) a Remak bundle (arrow) without any degenerating changes. Scale bar, 1 µm. in **b**, axons within Remak bundles in tibial nerves were enumerated. In **c**, axon diameters were measured. Results are shown as mean and s.e.m, with a minimum of 810 measurements made for each group. In **d**, Schwann cell diameters were measured. Results are shown as mean and s.e.m, with a minimum of 550 measurements made for each group. ***p-value < 0.0001

Having demonstrated that LepVax immunization preserved motor nerve function, we then determined its impact on sensory nerves. Electron microscopic examination of peripheral nerves revealed intact and fragmented *M. leprae* within myelinated axons, Remak Schwann cells and in axoplasm from infected unimmunized and LepVax immunized animals (Fig. 4a). In comparison to the axons from uninfected animals (Fig. 4a, panel i) the axons from infected armadillos exhibited intra axonal edema with loosening of axonal contents (Fig. 4a, panels ii and iii). Although an occasional degenerating Remak bundle and macrophages were identified in infected armadillos, no obvious lymphocytic cell infiltration was observed (Fig. 4a, panels iv, v and vi). Similar to humans, in armadillos the Remak bundles of tibial nerve predominantly carried single axons irrespective of group (Fig. 4b, uninfected: 44%, infected: 43%, immunized: 53%).^20^ Degenerating Remak Schwann cells were only rarely observed in either uninfected or infected, LepVax-immunized armadillos (<2%). This contrasted with the increase to 5% presence of empty denervated Remak in infected, unimmunized animals. In the uninfected animals, the axon was more compact (481.2 ± 7.4 nm) than those observed in *M. leprae* infected armadillos (Fig. 4c, *p*-value < 0.0001). *M. leprae* infection induced significant dilation of axons, although LepVax immunization significantly reduced this relative to unimmunized armadillos (infected immunized: 686.6 ± 10.17 nm versus infected unimmunized: 718.9 + 9.7 nm; *p*-value < 0.0001). Residency of *M. leprae* within the cytoplasm of Schwann cells was also implied by the slight, but proportionate, increase in the ratio of axons to Schwann cells in infected armadillos (unimmunized: 1:2.1 versus immunized: 1:2.3) relative to that observed in uninfected armadillos (1: 1.95). Remak Schwann cell were significantly larger in infected animals in comparison to the Schwann cells observed in uninfected armadillos (Fig. 4d, *p*-value < 0.0001) and, in contrast to axonal changes, the Schwann cell size in infected animals was not altered by LepVax immunization (Fig. 4d). No remarkable presence of degenerated Schwann cell processes, empty basal lamina tubes or fibroblastic proliferations were observed. Taken together, these data indicate that immunization with LepVax did not precipitate, but rather alleviated, *M. leprae* infection-induced damage to the tibial nerve.

### Characterization and enumeration of cutaneous nerves of distal leg

**Fig. 5:**
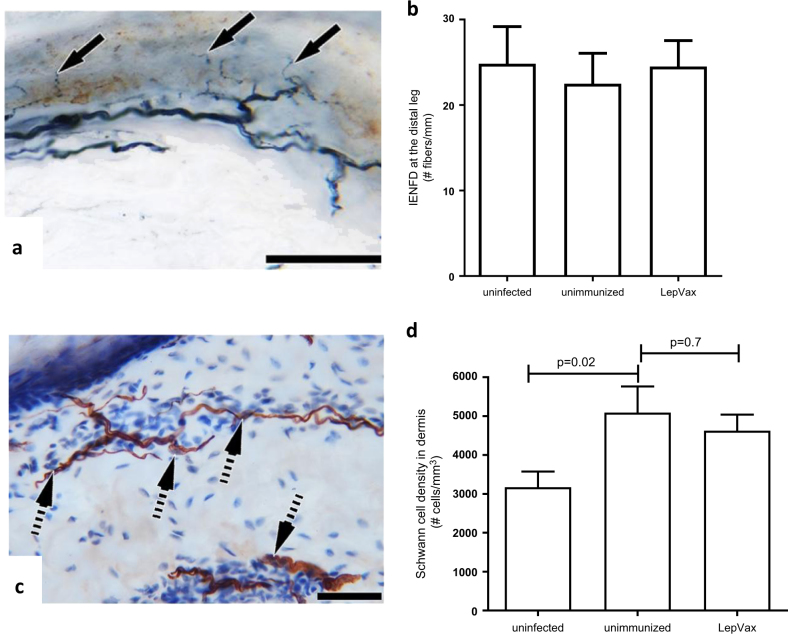
Morphometry of cutaneous nerves in armadillos. 3 mm skin punches were made at the distal leg in uninfected armadillos (*n* = 9) and in infected armadillos that were either unimmunized (*n* = 11) and LepVax-immunized (*n* = 11) at the 16th month after *M. leprae* innoculation. In **a**, the cutaneous innervation exhibits a comparable pattern to that observed in humans. In **b**, intraepidermal nerve fiber densities were measured. Results are shown as mean and s.e.m. In **c**, the brownish strands of Remak Schwann cell bundles in the dermis exhibiting Schwann cell nuclei (broken arrows) can be observed. In **d**, Schwann cells were enumerated using stereological optical fractionator probe. Results are shown as mean and s.e.m

The cutaneous innervation in *M. leprae*-infected armadillos followed a pattern comparable to that observed in leprosy patients, with the axons shredding the Schwann cell covering before entering the epidermis (Fig. 5a). In uninfected animals the distal skin exhibited dense epidermal nerve fibers and, regardless of if they had been immunized or not, the epidermal nerve fiber density was not significantly altered in *M. leprae*-infected animals (Fig. 5b). Remak Schwann cells of the cutaneous nerves in the distal leg were, however, denser in the infected armadillos in comparison to uninfected armadillos, suggesting that proliferation of the Schwann cells was occurring in response to *M. leprae* infection at cooler distal sites (Fig. 5c,d; *p*-value = 0.02). Immunization with LepVax slightly reduced the Remak Schwann cells density in the skin relative to unimmunized, infected armadillos, indicating that the vaccine certainly did not exacerbate sensory nerve involvement but rather provided some protective benefit to the cutaneous nerves.

## Discussion

Despite the impact of WHO-MDT on global prevalence, drug treatment of patients is unlikely to be sufficient to eliminate leprosy and further efforts are required to prevent its re-emergence. Targeting at-risk populations, amongst whom many individuals may already be infected with *M. leprae*, with an effective vaccine appears to be more a tenable long lasting strategy. The potential application of a vaccine under these circumstances has met with some reservations because neurological injury continues in patients even after the commencement of MDT and can be exacerbated during immune-mediated, inflammatory reactional episodes. It is therefore of particular importance to not only show that a vaccine can limit *M. leprae* infection but also does not exacerbate nerve damage. Our data identified antigens that can confer protection in a small animal system and this led to the generation of LepVax, a chimeric fusion protein composed of four distinct protein moieties in conjunction with the TLR4-based adjuvant formulation GLA-SE. Prophylactic immunization of mice with LepVax also significantly inhibited *M. leprae* outgrowth. Use of LepVax in the experimental post-exposure setting involving *M. leprae*-infected armadillos demonstrated not only its safe use but efficacy against motor nerve damage.

It is known that *M. leprae* infection in nine-banded armadillos (*Dasypus novemcinctus*), the only other host of *M. leprae* that closely recapitulates many of the structural, physiological, and functional aspects of leprosy observed in humans.^11–14,25^ Almost 70% of the armadillos manifest a lepromatous-type response to *M. leprae*.^16,17^ Monitoring studies of MB leprosy patients identified that approximately 40% had a reaction, neuritis, or new nerve function impairment event within 2 years following diagnosis that was detectable by monofilament testing and voluntary muscle testing.^26^ Nerve function was evaluated at each visit using nerve conduction, quantitative thermal sensory testing and vibrometry, dynamometry, and sub-clinical neuropathy was considered extensive (20–50%), even in patients who did not develop an outcome event. CMAP velocities were frequently affected, with velocity impaired in up to 43% of motor nerves. We have previously demonstrated that the distal limb in armadillos exhibit a length dependent type of innervation similar to that observed in patients, with denser innervation at the proximal sites than in the distal leg.^13^ Experimentally inoculated armadillos developed conduction abnormalities that manifested as depressed CMAP amplitude (<0.9 mV), or even total conduction block similar to observations in humans.^27^ Peripheral nerves are immune privileged due to the blood nerve barrier, and Schwann cells provide a safe niche for *M. leprae* survival, propagation and initial colonization, allowing for the long incubation period before manifestation of any clinical signs.^18,28,29^ Indeed, during lepromatous leprosy the sequence of sensory loss follows relative body temperatures with cooler areas providing the most favorable environment for *M. leprae* localization and proliferation.^30–34^ Experimental *in vitro* studies have shown that *M. leprae* can reprogram adult Schwann cells by altering host-gene expression, and the bacterially reprogrammed cells resemble progenitor/stem-like cells (pSLC) of mesenchymal trait. The pSLC acquire migratory and immuno-modulatory characteristics and release chemokines, cytokines and growth/remodeling factors and these reprogrammed cells potentially play a role in the initiation of neuropathogenesis during early *M. leprae* infection.^35,36^ Our data document a series of pathological processes showing that *M. leprae* survive within the superficially placed posterior tibial nerves and distal Schwann cells: the presence of *M. leprae* within edematous axons and Remak Schwann cells without any obvious inflammatory response, axonal degeneration and demyelination explicitly reflects the preclinical phase of silent neuritis in human leprosy. These edematous axons probably impede the nerve conduction that is observed by the early occurrence of depressed CMAP amplitude. The abnormal nerve conduction deficit elicited later in the course of the disease is consistent with the longstanding human leprosy neuritis. Interestingly, in addition, we noted that the distal dermal Remak Schwann cells undergo significant proliferation and this step precludes distal small sensory axonal loss, providing an apparently safe site for further bacterial propagation and colonization. To our knowledge these data provide the first documented pathological evidence that the pattern of peripheral neuropathy in armadillos can depict an early “pre-clinical silent neuropathy” model of leprosy.

In addition to implications with respect to the understanding of neuropathogenesis during leprosy, this is also the first report of post-exposure vaccination on *M. leprae*-associated neuropathy in a controlled system. Anecdotal reports, and now clinical evidence, indicate that BCG immunization may precipitate the onset of PB disease in some individuals, with speculation that infected but asymptomatic *M. leprae*-infected individuals are at the greatest risk.^37,38^ While a PB presentation is preferred over MB disease, the outcome is still damaging. Our studies demonstrate that BCG vaccination precipitates nerve damage in *M. leprae*-infected armadillos, supporting this hypothesis. The presentation was rapid and severe, with 3 of 11 infected animals immunized with BCG removed from the study within 12 months. In direct contrast, immunization with LepVax reduced and significantly delayed nerve damage. When LepVax-treated armadillos were compared with unimmunized armadillos, the CMAP was significantly improved and tibial nerve axons regained axonal size toward that observed in uninfected armadillos. It is noteworthy that siblings of the BCG-immunized animals that had to be removed were included in both the infected, unimmunized and infected, LepVax-immunized groups, and these siblings were monitored for over 2 years without observing any adverse events (data not shown). Thus, unlike BCG, post-exposure immunization with LepVax appears to be safe and does not induce damage to distal sensory nerve fibers in infected animals. It was also noteworthy that ID93/GLA-SE immunization did not alter the clinical course of the infection in armadillos. Although the reason for these differences between the vaccines is unclear, it is suggestive of differential antigen presentation by infected cells such that the antigen-specific immune response induced by LepVax, as compared to the responses elicited by either BCG or ID93/GLA-SE, allows for killing of *M. leprae* without inducing axonal edema or persistent local inflammation. The protection afforded by LepVax was not absolute and dissipated somewhat after several months, possibly due to the highly aggressive nature of the model involving intravenous inoculation of a large number of bacilli to invoke a lepromatous manifestation in armadillos. We believe that LepVax will retain its protective efficacy under less rigorous physiological conditions encountered in field conditions (lower exposure, more progressive advancement to high bacterial burdens, predilection to more borderline presentations, etc.). Clinical trials in various at-risk groups have the potential to address this hypothesis.

In conclusion, we have developed a single chimeric fusion protein that, when used in conjunction with the clinically approved GLA-SE formulation in a classical prophylactic immunization strategy, provides protection against *M. leprae* challenge of mice. Furthermore, by extending our studies into armadillos we were able to advance our understanding of leprosy *per se* by showing that *M. leprae* infection induces neuropathy that mimics the pre-clinical silent neuropathic situation that develops in leprosy patients. In stark contrast to BCG vaccination, the provision of LepVax after *M. leprae* infection (post-exposure immunoprophylaxis) was not only safe but efficacious, alleviating sensory nerve damage and both delaying and alleviating motor nerve damage in animals infected with high doses of *M. leprae*. Advancement of the LepVax vaccine to clinical testing appears prudent, with the potential to provide sustained, active protection against *M. leprae* infection. This represents a critical step toward the true elimination of leprosy.

## Materials and methods

### Ethics statement

Mouse experiments were conducted in accordance with procedures approved by either the Infectious Disease Research Institute (IDRI), National Institute of Infectious Diseases (NIID) or National Hansens Disease Program (NHDP) Animal Care and Use Committee, respectively. Armadillo experiments were conducted in accordance with procedures approved by the NHDP Animal Care and Use Committee. Animals were distributed randomly in different cages with group sizes calculated to allow minimal animal usage required to provide proper statistical analysis based on estimates of anticipated variation of rodent models of *M. leprae* infection and on prior experience with the experimental systems. The investigators were not blinded to analysis.

### Mice and immunizations

Female, 6–8 week old wild type C57BL/6 (B6) mice were purchased from Charles River Laboratories (Wilmington, MA). Mice were immunized with recombinant protein formulated with saline, SE or glucopyranosol lipid adjuvant (GLA)-SE, to provide a final protein concentration of 10 μg antigen and 20 μg GLA-SE.^39^ Mice were immunized up to three times by subcutaneous (s.c) injection of 0.1 ml volume at the base of the tail at 2-week intervals. Investigators were not blinded to the status of each treatment group. Mice were maintained in specific pathogen-free conditions and all procedures were approved by the pertinent institutional animal care and use committees (IDRI and NHDP).

### Antibody responses

Mouse sera were prepared following collection of retro-orbital blood into microtainer serum collection tubes (VWR International, West Chester, PA) followed by centrifugation at ~135×*g* for 5 min. Each serum was then analyzed by antibody capture ELISA. Briefly, ELISA plates (Nunc, Rochester, NY) were coated with 1 μg/ml recombinant antigen in 0.1 M bicarbonate buffer and blocked with 1% BSA-PBS. Then, in consecutive order and following washes in PBS/Tween20, serially diluted serum samples, anti-mouse IgG, IgG1 or IgG2c-HRP (all Southern Biotech, Birmingham, AL) and ABTS-H_2_O_2_ (Kirkegaard and Perry Laboratories, Gaithersburg, MD) were added to the plates. Plates were analyzed at 405 nm (EL_X_808, Bio-Tek Instruments Inc, Winooski, VT). Endpoint titer was determined as the last dilution to render a positive response, determined as two times the mean optical density of the replicates derived from sera from unimmunized mice in Prism software (GraphPad Software, La Jolla, CA).

### Antigen stimulation and cytokine responses

Single-cell suspensions were prepared by disrupting spleens between frosted slides. Red blood cells were removed by lysis in 1.66% NH_4_Cl solution, then mononuclear cells enumerated by ViaCount assay with a PCA system (Guava Technologies, Hayward, CA). Single-cell suspensions were cultured at 2 × 10^5^ cells per well in duplicate in a 96-well plate (Corning Incorporated, Corning, NY) in RPMI-1640 supplemented with 5% heat-inactivated fetal calf serum and 50,000 Units penicillin/streptomycin (Invitrogen). Cells were cultured in the presence of 10 μg/ml antigen for 72–96 h, after which culture supernatants were harvested and cytokine content assessed. IFNγ concentrations within culture supernatants were determined by ELISA performed according to manufacturer’s instructions (eBioscience) and optical density was determined using an ELx808 plate reader.

### Determination of bacterial burden in mice

To assess *M. leprae* growth, live *M. leprae* bacilli (Thai-53 strain) were purified from the footpads of *nu*/*nu* nude mice at NHDP or NIID. Mice were inoculated with 1 × 10^4^ bacilli by s.c. injection into each foot pad. Foot pads were harvested 12 months later and the bacilli were enumerated by direct microscopic counting of acid-fast bacilli according to the method of Shepard and McRae.^40^

### M. leprae infection of armadillos

The majority of the armadillos used in this study were genetically identical siblings that were allocated across the different treatment groups, thereby providing additional statistical power to the study. All armadillos were inoculated by intravenous injection with a suspension of 1 × 10^9^ viable *M. leprae* derived from passage in nude mice.^41^

### Characterization of peripheral nerves of armadillos by electron microscopy

The nerves were fixed for 48 h in 4% paraformaldehyde -3% glutaraldehyde and then transferred to 0.1 M Sorensen’s phosphate buffer. Sections were post-fixed with osmium, embedded in plastic and one-micron thick sections were made and stained with 1% toluidine blue. Thin 60–70-nm sections were mounted on Formvar-coated (Sigma-Aldrich) 50 mesh grids and stained with uranyl acetate (2.5% in 50% ethanol) and lead citrate (3%). The nerve sections were examined with a digital Zeiss Libra 120 electron microscope and Remak bundles identified using previously specified criteria.^42,43^ Point lists were randomly made at 4000 × to scan different sites in the sections and a minimum of 50 digital photographs were captured at a magnifications of 8000× to 16000×. Images were uploaded onto iTEM program (Olympus soft imaging), and Remak Schwann cells carrying unmyelinated axons were identified by specified criteria.^44,45^ An individual linear array was used to measure the mean axonal and Schwann cell diameter (nm, shortest distance) and the total number of unmyelinated axons per Remak Schwann cell quantified according to the established methodology.^20,46^

### Immunohistochemistry of skin sections

Skin biopsies were fixed in 2% paraformaldehyde/lysine/periodate for 24 h, sectioned with a sliding microtome into 50-µm thick, frozen, vertical, free-floating sections, which then were placed in 24-well tissue culture plates for immunostaining. Each skin section was incubated with the following primary antibodies diluted in blocking buffer: anti-PGP 9.5 (Chemicon, Temecula, CA), as a pan axonal marker, and mouse anti-p75 nerve growth factor receptor (Millipore), as a phenotypic marker for Schwann cells. Nonspecific binding of secondary antibodies was prevented by incubating sections with 4% normal goat serum diluted in 1.0% Triton X-100, 0.5% nonfat powdered milk in Tris-buffered saline (pH 7.4)]. Sections were rinsed in Tris-buffered saline (pH 7.4) and transferred to secondary antibodies. Secondary antibodies (diluted 1:100 in blocking buffer) included goat anti-rabbit IgG (Vector Laboratories, Burlingame, CA) and goat anti-mouse IgG (Vector Laboratories). The sections were then incubated with Avidin–Biotin Complex solution (Vector Laboratories), and color was developed with chromogens. PGP 9.5-stained sections were developed with blue/gray (Vector SG substrate) chromophore and p75-stained sections with diaminobenzidine. One percent eosin and Mayer’s hematoxylin were used as counterstains.

### Morphometry and histomorphometry of Schwann cells

Intra epidermal nerve fiber densities (IENFD) in skin biopsies were determined using established counting rules^19,47,48^ and IENFD was expressed as fibers/mm. Schwann cell bands within the dermis were identified in the skin sections by the presence of brown colored cytoplasmic staining by p75 in the setting of elongated blue-staining nuclei (counter stained with Mayer’s hematoxylin). The Schwann cell nuclei were identified and quantified using a design-based stereology methodology. The area of interest was defined as the dermal region extending from the epidermal/dermal junction to a dermal depth of 2000 µm. A contour was made at the area of interest under an ×2.5/0.075 Plan–Neofluor objective of a Zeiss light microscope and the cells were counted using ×63/1.40, oil Plan–Neofluor objective. A sampling grid 200 mm × 150 mm, with a dissector height of 15 mm and guard zones of 2 mm was used and the absolute numbers of Schwann cells were obtained using the “.DAT” files of optical fractionator probe of Stereo Investigator (MBF Bioscience).^49,50^ The Schwann cell density was determined as number of cells/mm^3^. The sampling design achieved a Gundersen coefficient of error 0.1. All morphometric measurements were conducted in a double-blinded, coded manner with no knowledge of the identifying data.

### Statistics

No statistical methods were used to ensure adequate power, but sample sizes were selected based on prior experience to achieve statistical significance with the lowest number of animals. The non-parametric Kruskal–Wallis analysis of variance test was used to compare the IFNγ levels among all of the groups. The *p*-values for studies resulting in normally distributed data including two groups were determined using the Student’s *t*-test. Where more than two groups were compared, *p*-values were attained by ANOVA analyses. Data were log-transformed for non-normal data sets prior to analysis. Statistics were generated using MS Excel (Microsoft Corporation, Redmond, WA) or Prism software (GraphPad Software, Inc., La Jolla, CA). Statistical significance was considered when the *p*-values were <0.05.

### Data availability

The underlying data reported in this paper are available from the corresponding author upon reasonable request.

## Electronic supplementary material

Supplemental Figure 1
