# Author Correction: LepVax, a defined subunit vaccine that provides effective pre-exposure and post-exposure prophylaxis of *M. leprae* infection

**DOI:** 10.1038/s41541-018-0055-7

**Published:** 2018-05-15

**Authors:** Malcolm S. Duthie, Maria T. Pena, Gigi J. Ebenezer, Thomas P. Gillis, Rahul Sharma, Kelly Cunningham, Michael Polydefkis, Yumi Maeda, Masahiko Makino, Richard W. Truman, Steven G. Reed

**Affiliations:** 1 Infectious Disease Research Institute, 1616 Eastlake Ave E, Suite 400, Seattle, WA 98102 USA; 2 National Hansens Disease Programs, Baton Rouge, LA USA; 3 Department of Neurology, Johns Hopkins University, Baltimore, MD 21209 USA; 4 Department of Microbiology, Immunology and Parasitology, LSU School of Medicine, New Orleans, LA USA; 5 Department of Mycobacteriology, Leprosy Research Center, National Institute of Infectious Diseases, Tokyo, Japan; 6 Department of Pathobiological Sciences, School of Veterinary Medicine, Louisiana State University, Baton Rouge, LA USA

**Correction to:** *npj Vaccines* (2018) 10.1038/s41541-018-0050-z, Published online 28 March 2018

A source of funding was mistakenly left out of the Acknowledgements. This research was also funded by NIAID/HRSA Interagency Agreement no. AAI15006-004-00000.

