# Supplementary material for: LepVax, a defined subunit vaccine that provides effective pre-exposure and post-exposure prophylaxis of M. leprae infection
Source: NPJ Vaccines. 2018 Mar 28;3:12. doi: 10.1038/s41541-018-0050-z (PMC5871809; doi:10.1038/s41541-018-0050-z)
Supplement: Supplementary file 1 — Supplemental Figure 1 [file 41541_2018_50_MOESM1_ESM.pptx]

## Slide 1
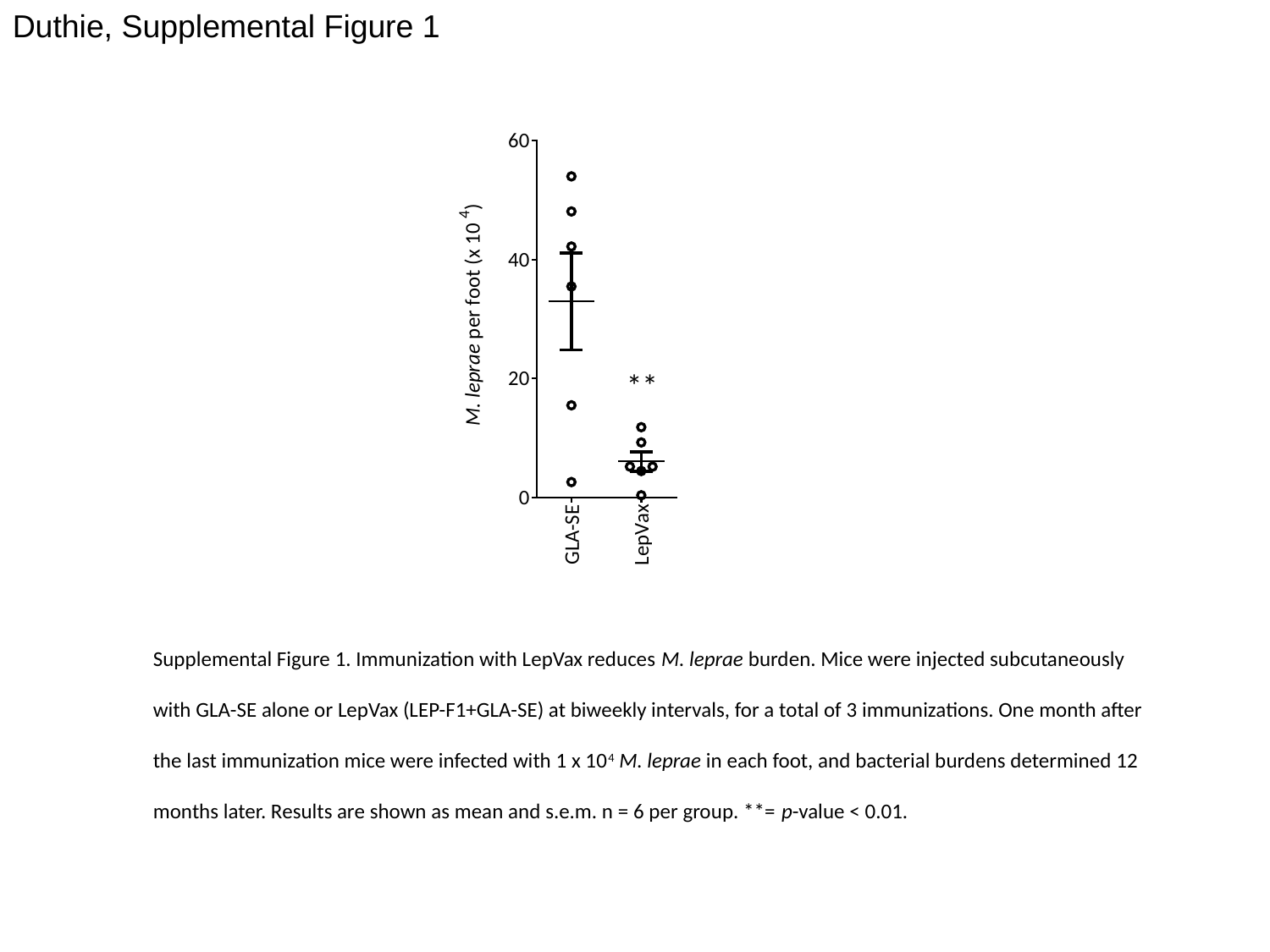

Duthie, Supplemental Figure 1
**
Supplemental Figure 1. Immunization with LepVax reduces M. leprae burden. Mice were injected subcutaneously with GLA-SE alone or LepVax (LEP-F1+GLA-SE) at biweekly intervals, for a total of 3 immunizations. One month after the last immunization mice were infected with 1 x 10^4^ M. leprae in each foot, and bacterial burdens determined 12 months later. Results are shown as mean and s.e.m. n = 6 per group. **= p-value < 0.01.
